# Supplementary material for: Flipping between Polycomb repressed and active transcriptional states introduces noise in gene expression
Source: Nat Commun. 2017 Jun 26;8:36. doi: 10.1038/s41467-017-00052-2 (PMC5484669; doi:10.1038/s41467-017-00052-2)
Supplement: Supplementary file 1 — Supplementary Information [file 41467_2017_52_MOESM1_ESM.pdf]

File Name: Supplementary Information
Description: Supplementary Figures and Supplementary Table

File Name: Peer Review File
Description:

A

	Mean number of reads (millions)	%
Mapped to exons	4.58	60.9
Mapped, no feature	1.74	23.2
Unmapped	1.19	15.9

B

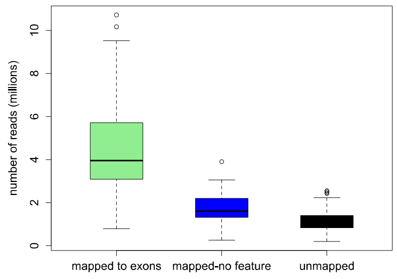

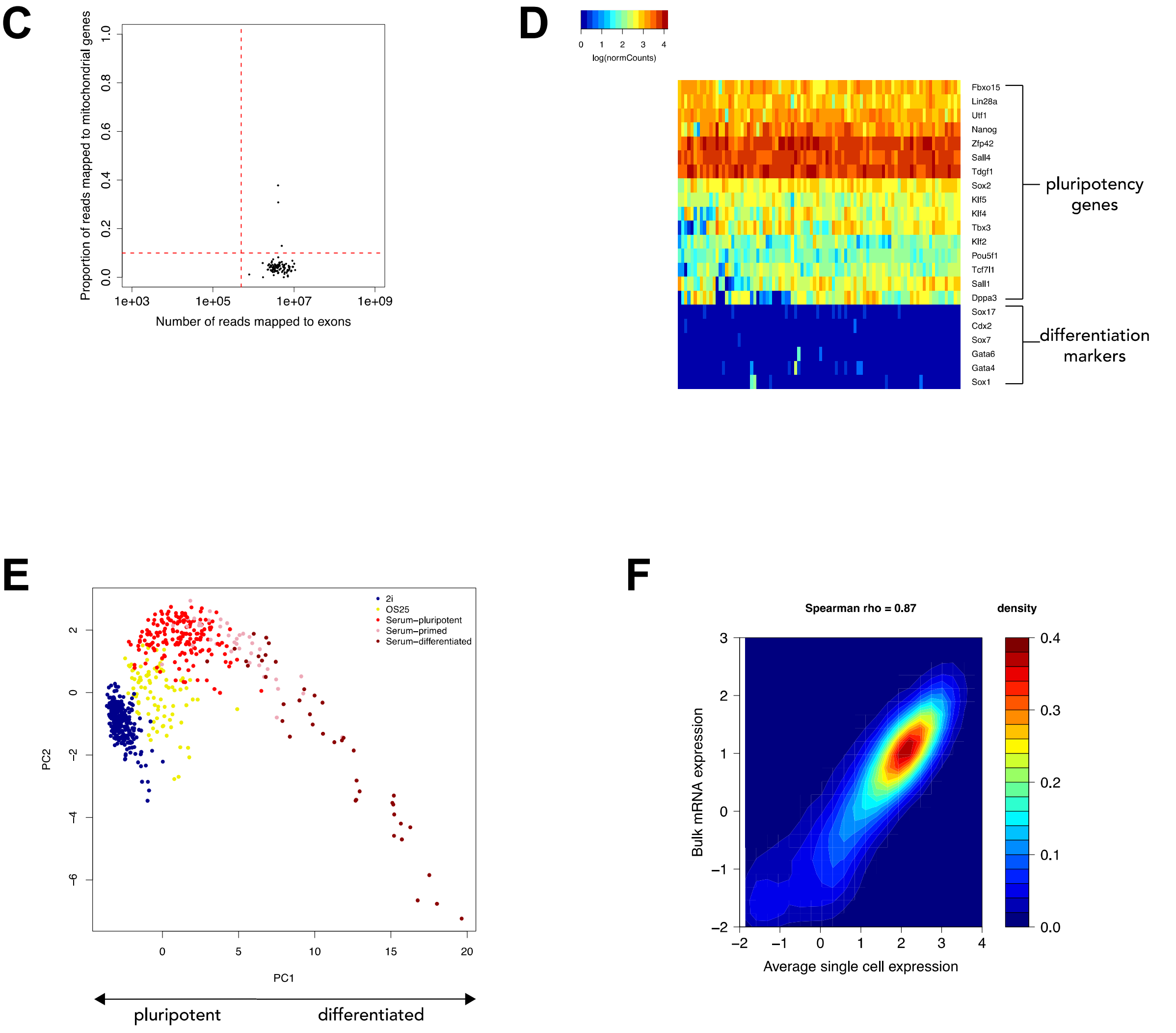

Supplementary Figure 1. (A,B) Single-cell data statistics: over 80% of reads were mapped to the *Mus musculus* genome (GRCm38) and over 60% to exons. (C) Quality control analysis for single cells. (D) Heatmap showing expression profiles of key pluripotency factors and differentiation markers in OS25 cells. There is homogenously high expression of pluripotency genes, and all differentiation markers are consistently “off”. This indicates that OS25 cells are all in a pluripotent state. (E) OS25 cells are shown together with other mESCs cultured in serum+lif and 2i from Kolodziejczyk et al. 2015. OS25 cells are more similar to the subpopulation of pluripotent serum cells, rather than the subpopulation of serum cells that are either “primed for differentiation” or “on the differentiation path”. (F) Average single-cell expression is highly correlated with bulk RNA-Seq (data from Brookes et al.[5]), Spearman’s correlation coefficient is 0.87.

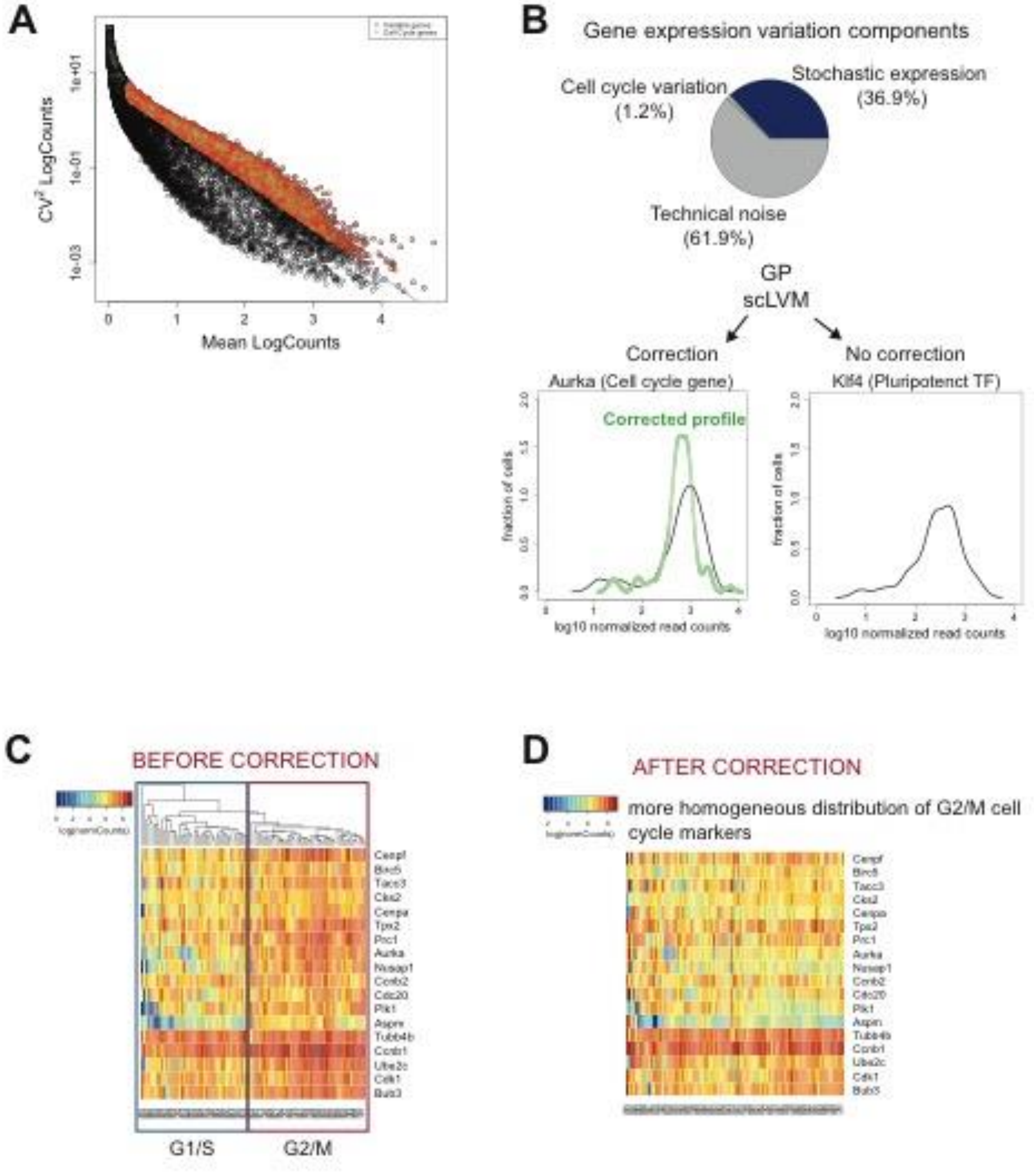

Supplementary Figure 2. (A) Squared coefficient of variation (CV^2) vs. average normalized read count of genes are shown (x and y-axes log10-scale). As gene expression levels increase, genes are more likely to show lower levels of variation. Variable genes are in red color and cell cycle genes (from Gene Ontology and Cycle base database) are in green color. (B) Gene expression variation components are unraveled by applying a recent method [13], which uses Gaussian Process Latent variable models in single-cells (scLVM). It is a two-step approach that first reconstructs cell cycle state and then uses this information to obtain "corrected" gene expression levels. Cell cycle contribution to variation is around 1% on average. In the lower panel, gene expression profiles for Aurka, a cell cycle gene and *Klf4*, a pluripotency transcription factor, are shown. After cell cycle regression, profile of Aurka becomes more homogeneous, whereas *Klf4* remains uncorrected. (C) Clustering of 90 cells based on cell cycle G2/M stage markers: there are two groups: one with high expression of G2 and M genes and the other with low expression of these genes. (D) Clustering after cell cycle correction: cell cycle effect is removed leading to more homogeneous expression distribution of these genes across the cells.

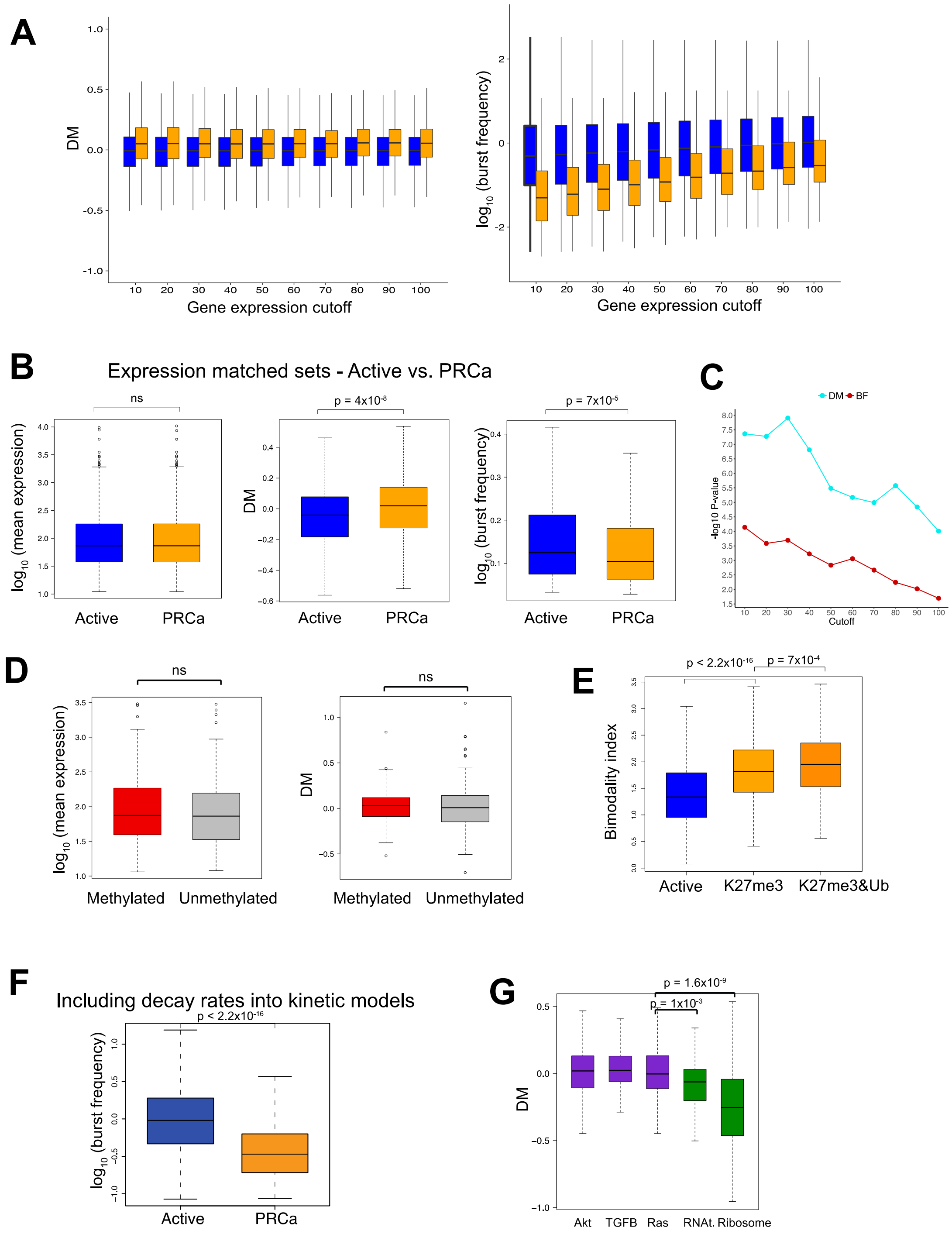

Supplementary Figure 3. (A) Distribution of DM and burst frequency levels across different cutoffs of gene expression. Two-sided Wilcoxon rank-sum test P-values for differences of DM between Active and PRCa genes are: $P<2.2x10^{-16}$, $P<2.2x10^{-16}$, $P<2.2x10^{-16}$, $P=6.7x10^{-15}$, $P=2.3x10^{-13}$, $P=1.8x10^{-13}$, $P=8.1x10^{-12}$, $P=1.7x10^{-13}$, $P=2.2x10^{-12}$ and $P=5.6x10^{-11}$ for gene expression cutoffs 10, 20, 30, 40, 50, 60, 70, 80, 90 and 100, respectively. For burst frequency levels, all P-values are $P<2.2x10^{-16}$. (B) Expression matched sets of Active and PRCa genes show that differences in DM and burst frequency levels are independent of gene expression levels. (C) –log10 P-values are shown for differences between DM levels and BF levels of expression-matched Active and PRCa groups across different expression cutoffs. The number of PRCa genes (that are expression-matched to same number of Active genes) are 666, 603, 540, 479, 427, 374, 341, 304, 280 and 262 for expression cutoffs of 10, 20, 30, 40, 50, 60, 70, 80, 90 and 100, respectively. (D) Comparison of gene expression variation profiles of methylated and unmethylated PRCa genes suggests that DNA methylation has no pronounced effect on transcriptional heterogeneity in mESCs. (E) PRCa genes are more bimodal than active genes. (F) Taking into account mESC degradation rates[21] and including them into our kinetic models does not result in major changes in kinetic parameters, thereby yields similar findings. (G) Median DM of KEGG signaling pathways PI(3)K-Akt, Ras signaling and TGF-beta signaling (shown in purple color) are significantly higher compared to median DM levels of pathways related to housekeeping functions, such as RNA transport and Ribosome (shown in green).

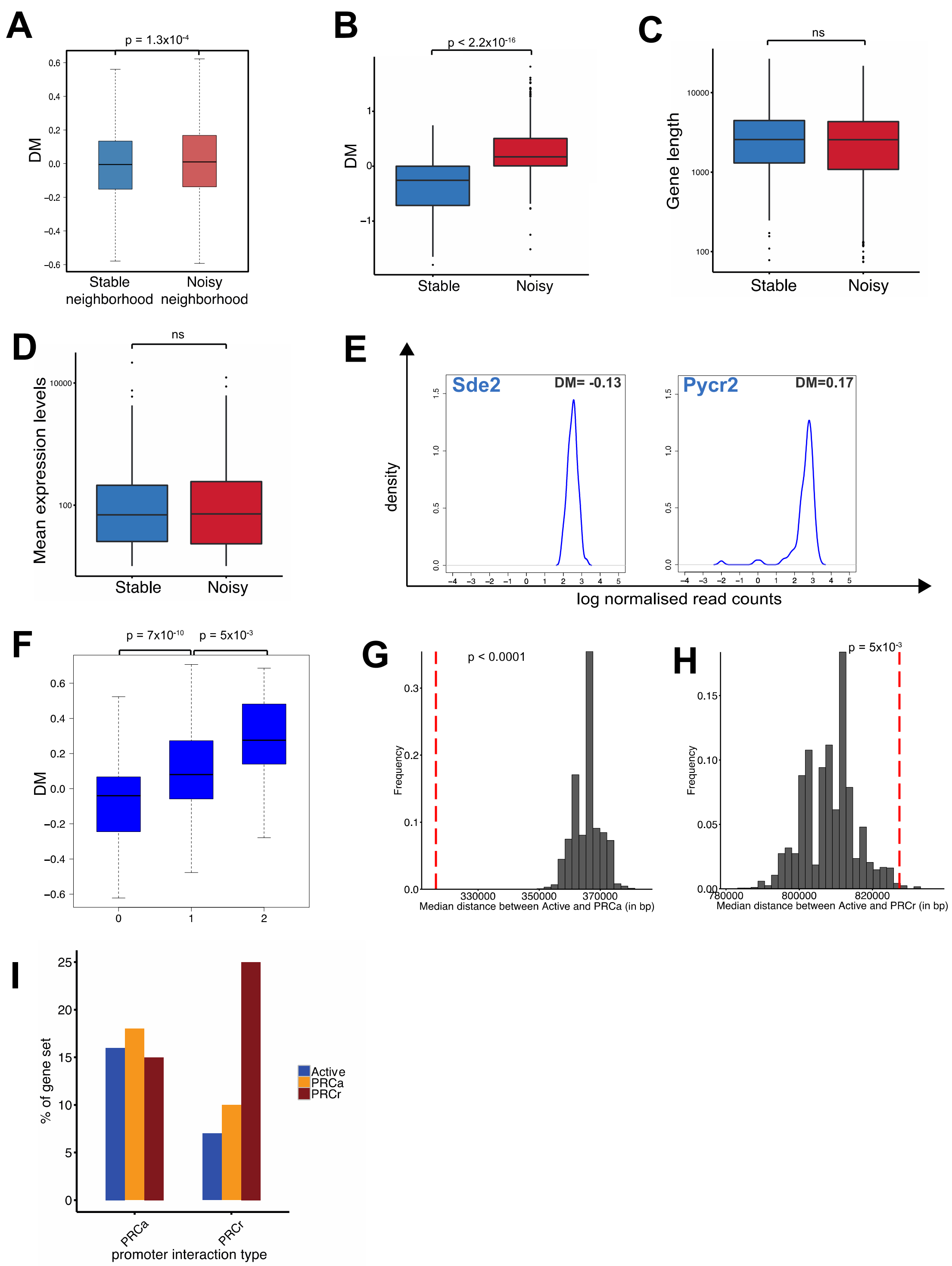

Supplementary Figure 4. (A) Noise levels of genes in the neighborhood of noisy genes are significantly higher than those of genes that flank stable genes. (B) The difference of DM between noisy and stable genes is significant ($P < 2.2x10^{-16}$ by two-tailed Wilcoxon rank sum test). (C) The difference of gene length between noisy and stable genes is not significant ($P = 0.1563$ by two-tailed Wilcoxon rank sum test). (D) The difference of mean expression levels between noisy and stable genes is not significant ($P = 0.8485$ by the two-tailed Wilcoxon rank sum test). (E) Gene expression profiles and DM levels of active genes; Sde2 and Pycr2 in one of the noisy clusters are shown. (F) Noise levels of active genes flanked by zero, one and two variable genes: genes flanked by two variable genes show highest levels of variation, while genes flanked by zero variable genes are more stable than other groups. (G) The observed median distance of Active genes to their nearest neighbor in the PRCa group, depicted by vertical dashed red line, are significantly less than expected by chance (empirical P < 0.0001). (H) The observed median distance of Active genes to their nearest neighbor in the PRCr group, depicted by vertical dashed red line, are significantly higher than expected by chance (empirical $P = 5x10^{-3}$). (I) Promoter preferences of gene sets: PRCr promoter preferences are different; PRCr genes are more likely to interact with PRCr promoters than PRCa genes (two-tailed Fisher's exact test $P < 2.2x10^{-16}$). Similarly, PRCa are more likely to interact with PRCr promoters than Active genes (two-tailed Fisher's exact test $P = 1x10^{-3}$)

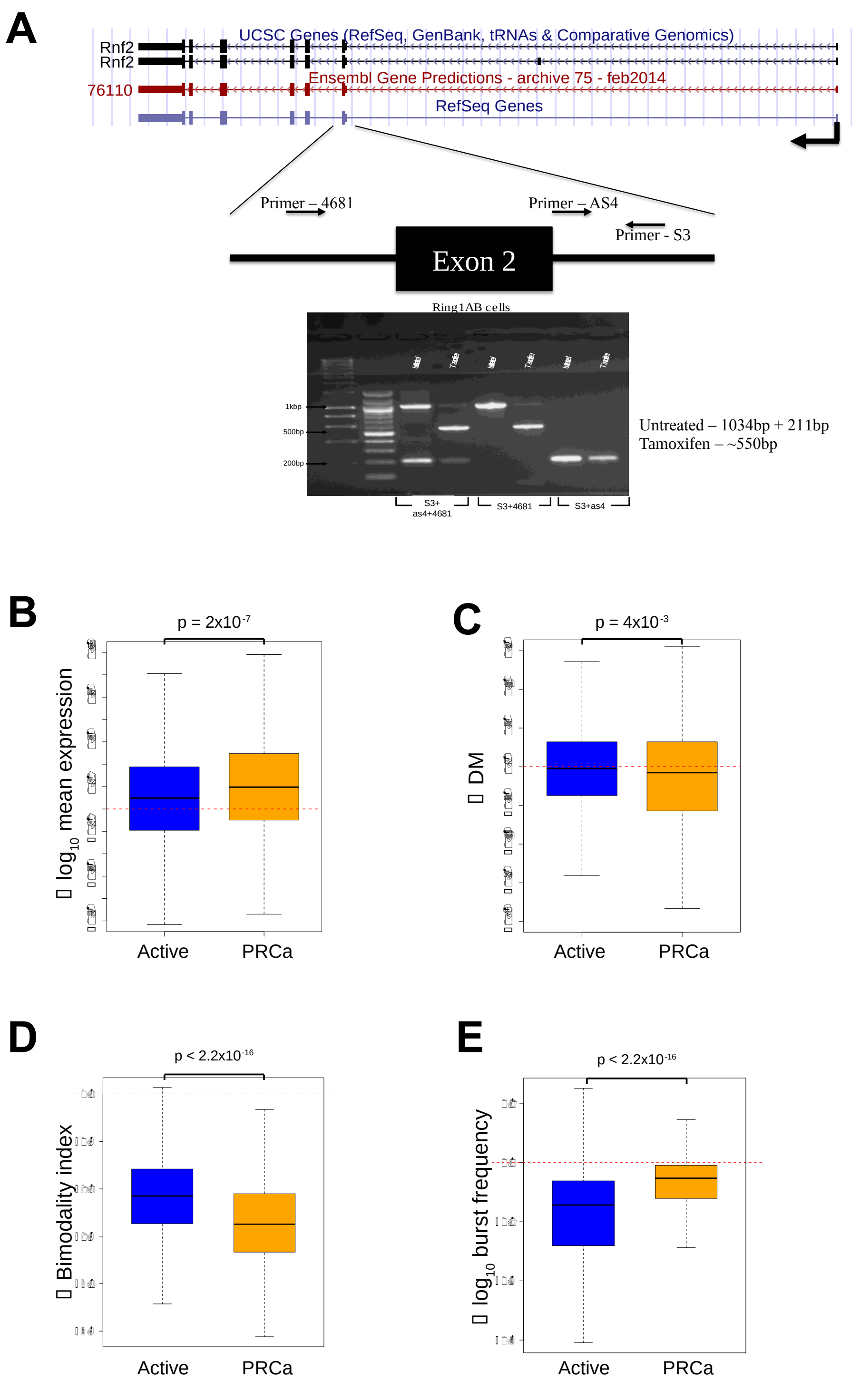

Supplementary Figure 5. (A) Schematic layout of Ring1B locus (UCSC mm10 reference assembly) and PCR primers to confirm Ring1b knockout. PCR amplification of genomic DNA from untreated (Ring1AKO) and tamoxifen-treated (Ring1ABdKO) to confirm Ring1B knockout are shown. Expected fragment size in untreated and Tamoxifen treated samples listed on right. (B) PRCa genes have a more pronounced change in mean gene expression and (C) noise levels (D) bimodality patterns than active genes. (E) Decrease in burst frequencies are more pronounced at active genes.

Supplementary Table 1. KEGG pathway enrichment of PRCa genes

**Pathways**	**FDR**
path:mmu04014 Ras signaling pathway	0.0002
path:mmu04060 Cytokine-cytokine receptor interaction	0.0010
path:mmu04151 PI3K-Akt signaling pathway	0.0023
path:mmu05206 MicroRNAs in cancer	0.0023
path:mmu05200 Pathways in cancer	0.0036
path:mmu04015 Rap1 signaling pathway	0.0043
path:mmu00604 Glycosphingolipid biosynthesis - ganglio series	0.0152
path:mmu05202 Transcriptional misregulation in cancer	0.0185
path:mmu04066 HIF-1 signaling pathway	0.0282
path:mmu04080 Neuroactive ligand-receptor interaction	0.0282
path:mmu04350 TGF-beta signaling pathway	0.0282
path:mmu04917 Prolactin signaling pathway	0.0422

KEGG pathways that show enrichment of PRCa genes are shown together and false discovery rates are reported.
